# Supplementary material for: Impact of the COVID-19 Pandemic Surveillance of Visceral Leishmaniasis in Brazil: An Ecological Study
Source: Infect Dis Rep. 2024 Feb 9;16(1):116–27. doi: 10.3390/idr16010009 (PMC10888456; doi:10.3390/idr16010009)
Supplement: Supplementary file 1 [file idr-16-00009-s001.zip › idr-2769417-supplementary.pdf]

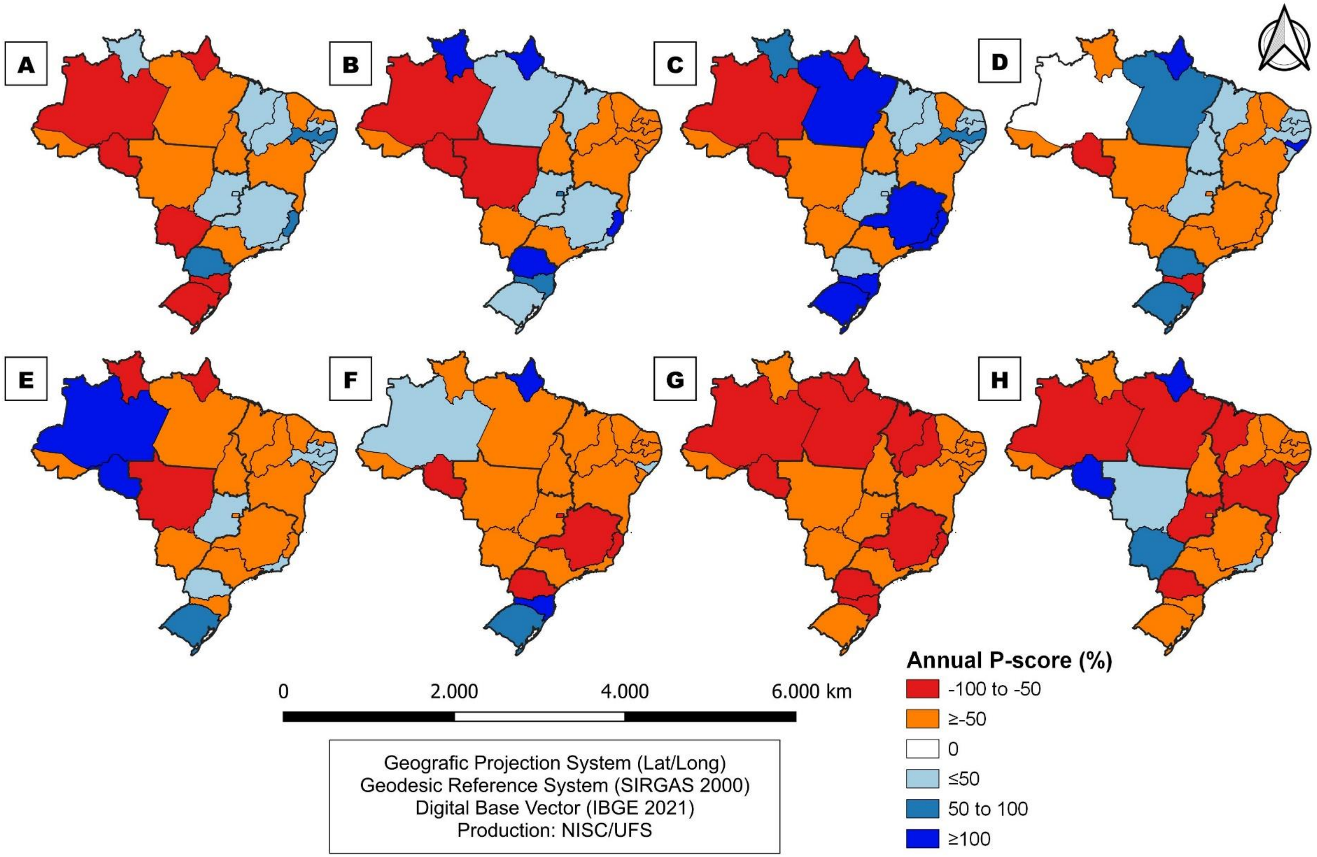

Figure S1. Spatial distribution of annual P-score of VL new cases notification. **A)** 2015. **B)** 2016. **C)** 2017. **D)** 2018. **E)** 2019. **F)** 2020. **G)** 2021. **H)** 2022.

Commented [SC1]: We changed to Figure S1 and Figure S2, please confirm.

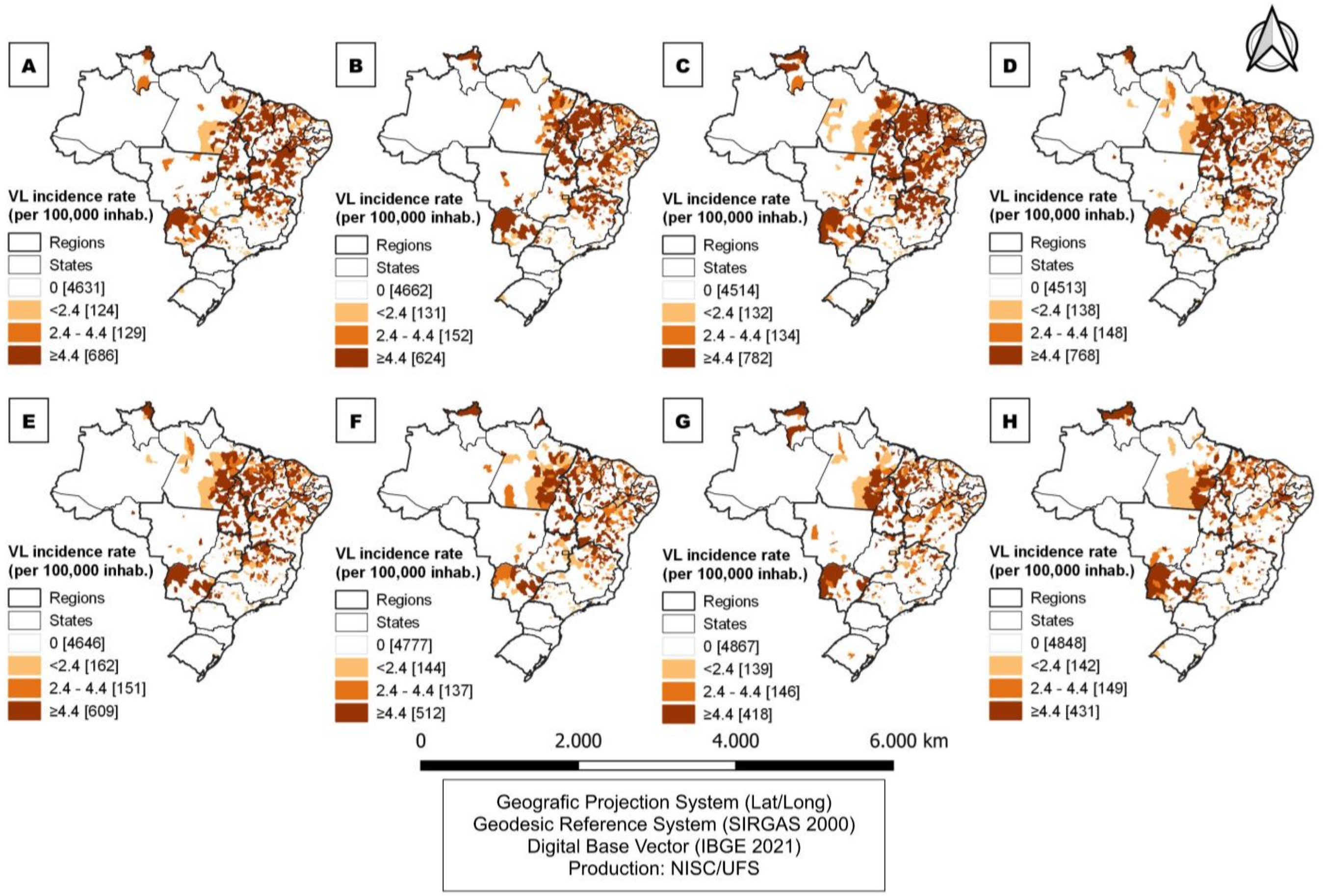

Figure S2. Spatial distribution of annual VL incidence. **A)** 2015. **B)** 2016. **C)** 2017. **D)** 2018. **E)** 2019. **F)** 2020. **G)** 2021. **H)** 2022.
